# Treatment Sequencing in a Chronic Lymphocytic Leukemia Patient with Central Nervous System Involvement

**DOI:** 10.1155/2018/7817918

**Published:** 2018-05-27

**Authors:** Filipa Mousinho, Tatiana Mendes, Paula Sousa e Santos, Maria João Acosta, José Pereira, Maria Arroz, Cândido Silva, Ana Paula Azevedo, Rita Oliveira, Martinha Chorão, Fernando Lima

**Affiliations:** ^1^Clinical Hematology Department, Hospital de São Francisco Xavier, Centro Hospitalar de Lisboa Ocidental, Lisbon, Portugal; ^2^Clinical Pathology Department, Flow Cytometry Laboratory, Hospital de São Francisco Xavier, Centro Hospitalar de Lisboa Ocidental, Lisbon, Portugal; ^3^Clinical Pathology Department, Hematology Laboratory, Hospital de São Francisco Xavier, Centro Hospitalar de Lisboa Ocidental, Lisbon, Portugal; ^4^Pathology Department, Hospital de Egas Moniz, Centro Hospitalar de Lisboa Ocidental, Lisbon, Portugal

## Abstract

Early-stage chronic lymphocytic leukemia (CLL) with neurologic involvement is a rare condition and should require a careful follow-up. Although no standard protocol exists for this condition, intrathecal chemotherapy, combined with systemic chemoimmunotherapy, has been used previously. This case describes the treatment of a patient with CLL and symptomatic compromise of the central nervous system. Our results suggest that a combination of chemotherapy, radiotherapy, and ibrutinib, administered sequentially over a 2-year period, led to a near-complete resolution of the cerebral spinal fluid neoplastic infiltration. Importantly, this response has been maintained with ibrutinib monotherapy for more than 12 months.

## 1. Introduction

B-cell chronic lymphocytic leukemia (B-CLL) is a chronic, long-term, and slowly developing leukemia characterized by a progressive accumulation of functionally incompetent monoclonal B lymphocytes. It is the most common form of leukemia found in adults of Western countries, but neurologic complications, arising from direct leukemic involvement of the central nervous system (CNS), are reported in only 1% of patients with B-CLL [1–3]. Although neurological symptoms can occur in patients with CLL, clinically significant CNS involvement (iCNS) by CLL cells is a rare condition [4]. The clinical manifestations of CLL with iCNS are heterogeneous and include headache, cranial nerve palsies, cerebellar signs, visual problems, and motor and/or sensory deficits. Imaging studies are neither specific nor sensitive in the detection of CLL involvement of the CNS, and the diagnosis is usually confirmed by flow cytometry (FC) analysis of the CSF.

Here, we describe the case of a patient with diagnosis of B-CLL who developed a neurologic impairment because of iCNS. The patient underwent treatment with schemes that included intrathecal chemotherapy, high-dose methotrexate, radiotherapy and ibrutinib, a Bruton tyrosine kinase (BTK) inhibitor. After nearly 26 months of follow-up, he had an almost complete response, with near-complete resolution of his neurologic symptoms.

## 2. Case Presentation

The 65-year-old man was diagnosed in 2006, with a B-CLL, with stage Rai 0/Binet A, and 13q deletion. He was kept under standard surveillance until significant disease progression with associated lymphocytosis. In January 2007, he started chlorambucil monotherapy as first-line treatment. This patient was treated for one year (12 cycles), achieving partial remission. In May 2015, this patient evidenced worsening of preexisting lymphocytosis, and later, on August 2015, it was also associated with palpable inguinal adenopathies. In September 2015, he was observed at the emergency hospital because of headache complaints, photophobia, vertigo, and extensive pain (from the cervical spine down to the inferior limbs). This pain led to an ataxic march that mimicked that of a cerebrovascular event, but the remaining neurologic examination was unremarkable.

**Figure 1:**
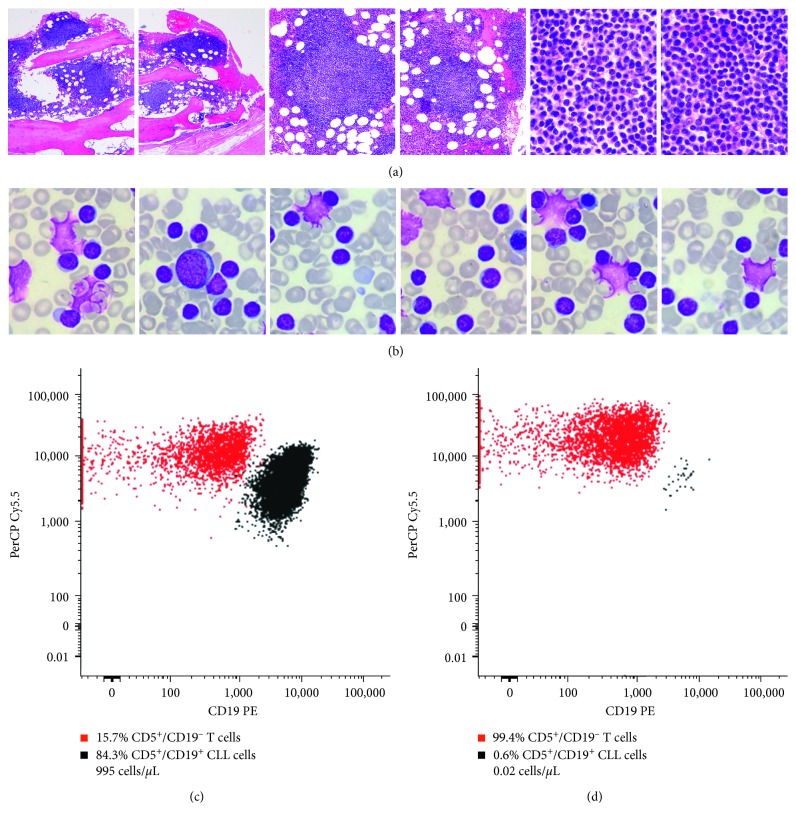
B-CLL characterization. (a) Bone marrow biopsy (HE): in September 2015, there was a 70% nodular and interstitial infiltration of small lymphocytes. (b) Bone marrow smear (MGG): in January 2016, numerous small lymphocytes, with clumped chromatin and scanty cytoplasm, smudge cells, and a few erythroblasts were detected. Dot plots illustrating cerebral spinal fluid (CSF), immunophenotyping CD5^+^/CD19^−^ T cells (red dots) and CD19^+^/CD5^+^ B-CLL cells (black dots) in October 2015 (c) and in August 2017 (d). Magnification: (a) ×40 (left), ×100 (center), and ×600 (right) and (b) ×100.

**Figure 2:**
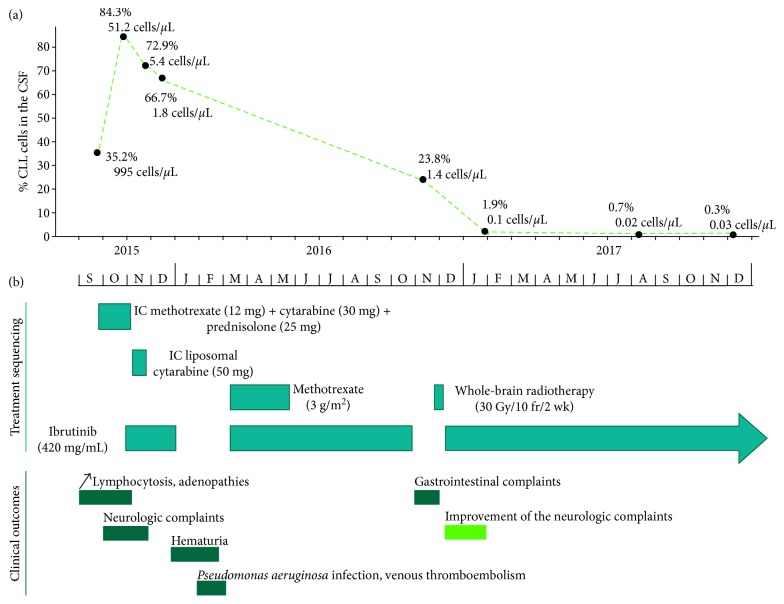
Evaluation of the CNS response and the clinical outcomes over time. (a) Flow cytometry analysis of the CSF over time, illustrating the % of CLL cells detected. The absolute number of counted CLL cells present in the diluted sample is shown underneath the percentage of cells detected, in dark grey. (b) Treatment course followed, illustrating therapy combinations and ibrutinib interruptions as well as the patient's clinical outcomes. Triple IC with methotrexate, cytarabine, and prednisone was administered 15 times, biweekly, but did not result in a positive response. Liposomal cytarabine was given 2 times (on the 10th and on the 24th of November 2015) but led to no response and might have led to the worsening of the neurologic symptoms due to a toxic myelitis. After resolution of the first toxicity (grade-2 hematuria), systemic high-dose methotrexate was administered together with ibrutinib. Whole-brain radiotherapy was administered (30 Gy/10 fr, in a regimen of 3 Gy/cycle/day, from the 24th of November until the 9th of December 2016) to decrease the CSF infiltration. After the first interruption, ibrutinib was restarted at 140 mg/day and then weekly increased to 280 mg/day and to 420 mg/day. After the second interruption, it was restarted at full dose (420 mg/day).

At that time, he was admitted to the hematology ward for characterization and management of the sudden neurologic impairment. We re-evaluated the disease, and a bone marrow study revealed a 70% CLL involvement, confirmed by FC, biopsy analysis (Figure 1(a)), and aspirate analysis (Figure 1(b)). In addition, cytogenetic evaluation using FISH confirmed the presence of a CLL clone with a 13q deletion, but no other anomalies were detected. In the context of CNS evaluation, the cerebral CT scan did not reveal any focal or diffuse lesions on the cerebral parenchyma. Further evaluation with magnetic resonance imaging (MRI) of the brain and spine was conducted with no evidence of acute signs to account for the symptoms. A diagnostic lumbar puncture was performed, and the cerebral spinal fluid (CSF) had normal glucose and protein levels. However, there was an elevated white blood cell count of 2.828 cells/*µ*L, with predominance of mononucleated cells. The FC analysis of the CSF revealed the presence of 35.2% of monoclonal B cells, 995 cells/*µ*L, compatible with B-CLL infiltration (Figure 2). The cytogenetic analysis of the CSF was made using fluorescence in situ hybridization (FISH) and was also positive for del(13q14.3) in 88% of the analyzed nuclei. These findings were suggestive of CLL involvement of the CNS, and we started a second-line therapy consisting of a BTK inhibitor combined with intrathecal chemotherapy (IC).

While awaiting approval for the targeted oral therapy, ibrutinib, the patient started IC. From September to November 2015, a total of 17 IC administrations were performed (15 administrations of methotrexate, cytarabine, and prednisolone, twice a week followed by 2 doses of liposomal cytarabine every 2 weeks). Nevertheless, no response was achieved, and there was a progressive worsening of the neurological deficits, with the development of paraplegia. We suspect that, at that stage, the worsening of the neurological condition was, at least in part, due to an eventual toxic myelitis related to IC administrations.

During IC treatment, in October 2015, CSF re-evaluation by FC revealed an infiltration degree of 84.3% (Figures 1(c) and 2). By the end of October 2015, the patient started ibrutinib, at 420 mg/day, and after the first week of treatment, the adenopathies disappeared. Nonetheless, two months later, ibrutinib had to be temporarily suspended due to the onset of grade-2 hematuria. This adverse event was resolved within two months, and therapy was re-introduced in a safe way.

On December 2015, the FC analysis of the CSF showed persistent neoplastic infiltration of about 66.7% and motivated the association of high-dose methotrexate, every 15 days, which was administered for a total of 6 cycles, until May 2016. Treatment with methotrexate plus ibrutinib was delayed until the beginning of March because of the onset of a *Pseudomonas aeruginosa* infection and because of an episode of venous thromboembolism. The combination of high-dose methotrexate with ibrutinib seems to have led to a decrease in the % of CNS infiltrating CLL cells from 66.7% to 23.8% (Figure 2).

Later that year, due to gastrointestinal complaints (related with intestinal pseudo-occlusion), ibrutinib treatment was suspended for another month. These complaints were not attributable to other etiologies, and after resolution, ibrutinib was re-introduced at full dose in December 2016. Meanwhile, because there was still a 23.8% CLL infiltration of the CSF, CNS radiotherapy was delivered, in November 2016, as denoted in Figure 2. Since then, ibrutinib therapy has been maintained, without further interruptions or dose reductions, and CSF analysis by FC has shown a continuous decrease in the number of infiltrated CLL cells (towards a minimum of 0.3% on the last analysis, Figure 1(d)).

Over the follow-up of 26-month treatment, there was almost a complete improvement of all neurologic deficits, and at the time of the last lumbar puncture, the patient presented only a small degree of paraparesis. Physical rehabilitation was commenced and has been contributing to an improvement of the paraparesis. Finally, there is still no evidence of splenomegaly or adenopathies, and the patient is continued to be treated daily with ibrutinib.

## 3. Discussion

Patients with early-stage, long-standing, or advanced CLL disease may experience localized leptomeningeal disease. However, a leptomeningeal dissemination of CLL cells in patients with early-stage disease is extremely rare [5], making it difficult to distinguish between CLL involvement and other etiologies [6]. The optimal treatment course for patients who have CLL with iCNS is unclear. The majority of them undergo treatment regimens that include IC with or without radiation therapy or receive systemic chemotherapy. For IC, common systemic drugs, that penetrate the blood-brain barrier (BBB), such as methotrexate, cytarabine, and corticosteroids, can be administered alone or in combination [7]. Also, several studies have demonstrated that ibrutinib crosses the BBB in patients with B-cell malignancies [8, 9] and can achieve a response in sensitive cases with iCNS. Nevertheless, because there are not many cases described, no treatment protocols have been established for CLL with iCNS.

In this case, aggressive regimens, such as those including fludarabine, have been excluded due to the patient's age and, especially, due to other comorbidities (severe pulmonary tuberculosis, in 2008, submitted to right upper lobectomy, with mixed ventilatory sequelae and a hypertensive cardiomyopathy). Hence, it seemed to us that IC with ibrutinib was an option with lower toxicity, with a lower risk of pulmonary infections, and easier to handle without delays between cycles, thus providing greater comfort to the patient.

After more than 2 years of combined treatment with chemotherapy, radiotherapy, and ibrutinib, the patient had a near-complete resolution of the CSF infiltration, which has been maintained with ibrutinib monotherapy for 12 months. This suggests that this treatment sequencing may benefit patients in similar cases when there is a neoplastic CLL infiltration of the CNS. Lastly, we believe that continuous treatment with ibrutinib may progressively improve the clinical outcomes of this patient and help to maintain a durable CNS response.
